# Abnormalities of brain gray matter in bipolar depression and unipolar depression

**DOI:** 10.3389/fpsyg.2025.1534065

**Published:** 2025-11-13

**Authors:** Li Zhou, Guowei Wu, Xinchun Li, Chang Liu

**Affiliations:** ^1^Department of Psychiatry, The Second People’s Hospital of Hunan Province (Brain Hospital of Hunan Province), Changsha, China; ^2^Department of Psychiatry, First Affiliated Hospital of Jinan University, Guangzhou, Guizhou, China; ^3^Mental Health Institute, Second Xiangya Hospital, Central South University, Changsha, Hunan, China

**Keywords:** depression, structural magnetic resonance imaging, gray matter volume, bipolar depression, unipolar depression

## Abstract

**Objective:**

We sought to investigate differences in gray matter (GM) volume among patients with bipolar depression (BD), unipolar depression (UD), and healthy controls (HCs) to identify distinct gray matter volume abnormality patterns that may help differentiate BD from UD.

**Methods:**

In total, 23 patients with BD, 22 patients with UD, and 24 HCs were recruited to undergo dimensional, structural magnetic resonance imaging. Using voxel-based morphometry (VBM), we investigated gray matter volume across the three groups.

**Results:**

Both patient groups showed significantly reduced gray matter volume in the right superior temporal gyrus and right insula compared to the HCs. Moreover, the right posterior cingulate cortex exhibited decreased gray matter volume in the BD group but increased gray matter volume in the UD group. In addition, gray matter reductions in the right superior temporal gyrus and right insula were significantly positively correlated with the total Hamilton Depression Rating Scale (HAMD) score in the UD group.

**Conclusion:**

Patients with BD and UD demonstrate different patterns of gray matter abnormalities in the right posterior cingulate cortex, which might be a biomarker for differentiating these two depressive disorders.

## Introduction

Bipolar disorder is a disorder that is characterized by recurrent episodes of mania and depression (Phillips and Kupfer, 2013; Green et al., 2007). Epidemiological studies suggest that bipolar disorder affects up to 5% of the general population. However, only 20% of individuals with Bipolar disorder experiencing a depressive episode receive the correct diagnosis within the first year of seeking appropriate treatment (Hirschfeld et al., 2003), and the average duration of misdiagnosis for Bipolar disorder exceeds 5 years (Phillips and Kupfer, 2013). Misdiagnosing Bipolar disorder as unipolar disorder is not rare clinically. Close to 60% of patients with Bipolar disorder are originally diagnosed with unipolar disorder depression (de Cardoso Almeida and Phillips, 2013; Hirschfeld et al., 2003). The phenomenon of misdiagnosing Bipolar disorder as unipolar disorder depression can lead to serious problems, including inappropriate medication—prescribing antidepressants for Bipolar disorder—which may, in turn, exacerbate symptoms (Berk et al., 2006) and increase the difficulty of treatment. However, distinguishing between Bipolar disorder and unipolar disorder depression is not easy clinically. Current diagnostic systems are based on symptomatology rather than etiology, and the higher prevalence of depressive symptoms relative to hypomanic symptoms during the course of Bipolar disorder and the presence of subthreshold manic symptoms during a depressive episode (Bowden, 2010; Bowden, 2001) result in misdiagnosis of the illness as unipolar disorder. However, it is unknown whether there are specific pathophysiological mechanisms that can differentiate these two depressive disorders.

Many studies using neuroanatomy, neurochemistry, and functional magnetic resonance imaging (fMRI) have shown neurobiological correlates of Bipolar disorder and unipolar disorder. Morphometric neuroimaging studies comparing patients with bipolar and unipolar depression with healthy controls (HCs) suggest the presence of neuronal abnormalities in these two mood disorders. For instance, voxel-based morphometry (VBM) studies have demonstrated that patients with unipolar depression show decreased gray matter (GM) density in the frontal cortex, temporal gyrus, and anterior cingulate cortex, along with increased gray matter density in the right insula and left amygdala (Kessler et al., 2003; Cerqueira et al., 2007). Moreover, gray matter abnormalities in the frontal cortex are associated with the severity of depressive symptoms (Matsuo et al., 2006; Chen et al., 2007). In parallel, the most consistent morphological finding in bipolar depression is the abnormally decreased volume of the frontal cortex relative to healthy controls (Kalmar et al., 2009). In addition, some studies have suggested increased gray matter volume in the amygdala (Frangou , 2005), which contrasts with findings in unipolar disorder (Phillips et al., 2003). A recent few studies have utilized structural magnetic resonance imaging to examine differences in brain structures between Bipolar disorder and unipolar disorder. In Bipolar disorder, increased gray matter volume has been reported in the anterior cingulate cortex, caudate, and putamen, while decreased gray matter volume has been reported in the prefrontal cortex, median cingulate cortex, hippocampus, and amygdala in unipolar depression (Matsuo et al., 2006; Cordes et al., 2001; Phillips et al., 2003; Greicius et al., 2004). Functional magnetic resonance imaging directly compared unipolar disorder and bipolar depression to examine differences in brain activation. For instance, Almeida et al. (2010) reported increased amygdalar activation in individuals with bipolar disorder compared to those with unipolar depression, when presented with mild sad and neutral facial expressions. Another study found that individuals with bipolar disorder exhibited reduced effective connectivity between the bilateral amygdala and ventromedial prefrontal cortex (VMPFC) in response to happy faces (Almeida et al., 2009). In parallel, the most consistent resting-state fMRI finding is the presence of distinctive patterns of functional connectivity (FC) abnormalities that differentiate unipolar and bipolar depression. In patients with unipolar disorder, increased activity has been observed in the thalamus and sub-cingulate at resting states (Greicius et al., 2007); and abnormalities in patterns of the fronto-limbic neural activity (Yurgelun-Todd et al., 2000; Anand et al., 2005).

These observed neuronal deficits in Bipolar disorder, which differ from those in unipolar disorder, may have potential value in distinguishing between the two disorders. However, existing studies have a major shortcoming: Patients with Bipolar disorder often included individuals in manic, depressive, or remission phases, which cannot rule out the interference of various disease states. Previous structural magnetic resonance studies on unipolar depressive disorder have suggested that factors such as depressive state (acute phase or remission phase), severity, age, and sex can all influence abnormal changes in local morphological structures (Lorenzetti et al., 2009). Furthermore, studies have found that long-term treatment with medications such as antidepressants and mood stabilizers exerts an effect on changes in local brain structures (Takahashi et al., 2010); for instance, lithium salts and antidepressants may lead to changes in gray matter volume in regions including the hippocampus, amygdala, and cerebellum (Gowen and Miall, 2007; Savitz et al., 2010). In our study, the influence of the aforementioned confounding factors on local brain morphological structures was controlled to a certain extent using strict enrollment criteria. We enrolled patients with unipolar and bipolar depressive disorders who had been taking medications for no more than 2 weeks, were in the acute phase of a major depressive episode during the MRI scan, and had a score of more than 17 on the Hamilton Depression Rating Scale. Using VBM, we compared differences in gray matter volume between these two disorders and investigated correlations between clinical data and gray matter volume in regions with significant group differences.

## Methods

### Study participants and recruitment

In total, 23 patients with bipolar depression and 22 patients with unipolar depression were recruited from the inpatient and outpatient units of the Department of Psychiatry, the Second People’s Hospital of Hunan Province (Brain Hospital of Hunan Province), Changsha, China. All patients were diagnosed with bipolar depressive disorder or major depressive disorder according to the Structured Clinical Interview for DSM-IV, Patient Version. The inclusion criteria were as follows: age between 18 and 45 years, Han Chinese ethnicity, completion of at least ninth-grade education or higher, sufficient comprehension and expressive capacity, a total score of ≥17 on the 17-item Hamilton Depression Rating Scale (HAMD), and a total score of <6 on the Young Mania Rating Scale (YMRS). The exclusion criteria were as follows: severe learning disability, current diagnosis of substance use disorder, alcohol consumption within 24 h prior to the interview or scanning, history of brain trauma or neurological disease, left-handedness, previous electroconvulsive therapy, and any other contraindications to MRI. All participants were on antipsychotic medication at the time of the study. Benzodiazepine treatment, if any, was discontinued at least 24 h prior to scanning. A total of 24 healthy controls were recruited from the Changsha city area. The inclusion and exclusion criteria were the same as those for patients with depressive disorders, except that controls should not meet the DSM-IV criteria for any Axis I psychiatric disorder. The samples for this dataset were mainly collected between January 2017 and December 2018. All patients with BP underwent MRI scanning within 30 min of being assessed with the HAMD and the YMRS. Healthy controls were recruited through advertisements at various universities in Changsha. Healthy controls who met the DSM-IV criteria for any Axis I psychiatric disorder were excluded. All HCs were well matched with the two patient groups in terms of sex (*χ*^2 = 1.003, *p* = 0.606), years of education (*t* = 0.423, *p* = 0.657), and age (*t* = 2.183, *p* = 0.121). The two patient groups were well matched in the HAMD scores. All participants provided informed consent to participate in the study. The study was approved by the ethics committee of the Second Xiangya Hospital of Central South University.

### Assessments and procedures

All participants were assessed for cognitive function using the Information and Digit Symbol Coding subsets of the Wechsler Adult Intelligence Scale (WAIS). Demographic data, including age, sex, and years of education, were recorded. Clinical information, including diagnosis and duration of illness for patients, was also recorded. All patients were assessed using the Hamilton Depression Rating Scale (HAMD; Hamilton, 1960) and the Young Mania Rating Scale (YMRS; Young et al., 1978). All patients underwent MRI within 1 week of diagnosis. Moreover, before the scan, all patients were assessed using the 17-item Hamilton Depression Rating Scale (HAMD) and the Young Mania Rating Scale (YMRS) to ensure that they were in a depressive episode at the time of scanning.

### fMRI data acquisition

Imaging data were collected using a 3.0 Tesla Philips Achieva whole-body MRI scanner (Philips, The Netherlands) with a gradient-echo echo-planar imaging (EPI) sequence. The acquisition parameters were as follows: repetition time [TR] = 7.5 ms, echo time [TE] = 3.7 ms, flip angle = 8°, matrix 64 × 64, slice thickness = 1 mm, gap = 0 mm, and slices = 180.

### Image processing

All images were visually inspected for artifacts or structural abnormalities before voxel-based morphometry (VBM) analysis was applied to the structural magnetic resonance imaging (MRI) data using SPM8 (Wellcome Trust Centre for Neuroimaging, Institute of Neurology, UCL, London, United Kingdom).^1^ The detailed steps of the VBM analysis were as follows. First, the origin of all structural images was manually set to the anterior commissure. Second, all images were segmented into gray matter (GM), white matter (WM), and cerebrospinal fluid (CSF) and imported into a rigidly aligned space (Zeng et al., 2014). Third, the segmented images were registered using the Diffeomorphic Anatomical Registration Through Exponentiated Lie algebra (DARTEL) toolbox (Van Dijk et al., 2012). This procedure generated a template for the group of individuals. Fourth, the resulting images were spatially normalized to the MNI space using affine spatial normalization. An additional processing step involved multiplying each spatially normalized image by its relative volume before and after normalization for the purpose of preserving the total amount of each tissue. Finally, the images were smoothed with an 8-mm full width at half maximum (FWHM) isotropic Gaussian kernel. After affine spatial normalization, the blood-oxygenation-level–dependent (BOLD) signal of each voxel was first detrended and then passed through a band-pass filter (0.01–0.08 Hz) to reduce low-frequency drift and high-frequency physiological noise. Finally, nuisance covariates, including head motion parameters, global mean signals, white matter signals, and cerebrospinal fluid signals, were regressed out from the BOLD signals.

### Voxel-based analysis

To investigate alterations in gray matter volume in the identified brain regions among the patients with BD, those with UD, and healthy controls, analysis of variance (ANOVA) was conducted on the smoothed images to compare the three groups. Between-group comparisons of gray matter volume were performed using two-sample *t*-tests on the smoothed images. Between-group differences were assessed using two-sample *t*-tests among the three groups [*p* < 0.05 with false discovery rate (FDR) correction, and uncorrected *p* < 0.001].

### Correlation analysis

To explore whether the GM abnormalities in the BD and UD groups were correlated with the severity of depressive symptoms, voxel-based correlation analyses were conducted between all voxels in the GM abnormal areas and the HAMD total scores. Pearson analysis was used to evaluate the relationship between gray matter volume showing group differences and the HAMD scores. Using the AlphaSim program,^2^ the resulting statistical map was corrected for multiple comparisons, with a significant level of *p* < 0.05 (a minimum cluster size of 10 voxels). One-sided *t*-tests (both left-sided and right-sided analyses) were applied to these samples. Only functional connectivities with (uncorrected) a *p*-value of <0.01 were retained for further analyses.

## Results

### Demographics, clinical, and behavioral data

Demographic information and clinical characteristics are presented in Table 1. There was no significant difference in sex and years of education among the three groups. The patient groups did not differ significantly in the HAMD scores or medication dosage. However, the UD group had a longer illness duration and was older than the BD group. In addition, the BD group showed significantly lower scores on the WAIS Digit Symbol subset relative to the UD group.

**Table 1:** Demographic and clinical characteristics (mean ± standard deviation).

Group	Healthy controls (*N* = 25)	Bipolar depression patients (*N* = 23)	Unipolar depression patients (*N* = 23)	F/T	*p*
	Mean (SD)	Mean (SD)	Mean (SD)		
Age (years)	27.72 (6.49)	27.22 (4.72)	31.04 (8.58)	2.183	0.121
Education (years)	13.00 (2.80)	13.37 (2.96)	12.61 (2.69)	0.423	0.657
Illness duration (months)	-	53.74 (49.67)	37.21 (44.62)	1.409	0.242
WAIS Information	19.29 (5.22)	14.58 (3.59)	17.56 (5.79)	7.459	0.001
WAIS Digit Symbol	79.07 (11.44)	53.56 (12.08)	69.50 (14.37)	27.365	<0.001
HAMD	-	21.57 (4.67)	20.05 (5.78)	0.506	0.671
YMRS	-	15.84 (7.7)	14.29 (6.53)	1.084	0.841
Sex	13 Male	11Male	13 Male	χ^2^	*p*
	12 Female	12Female	10 Female	1.003	0.606

### Comparison of whole-brain gray matter volumes

**Figure 1:**
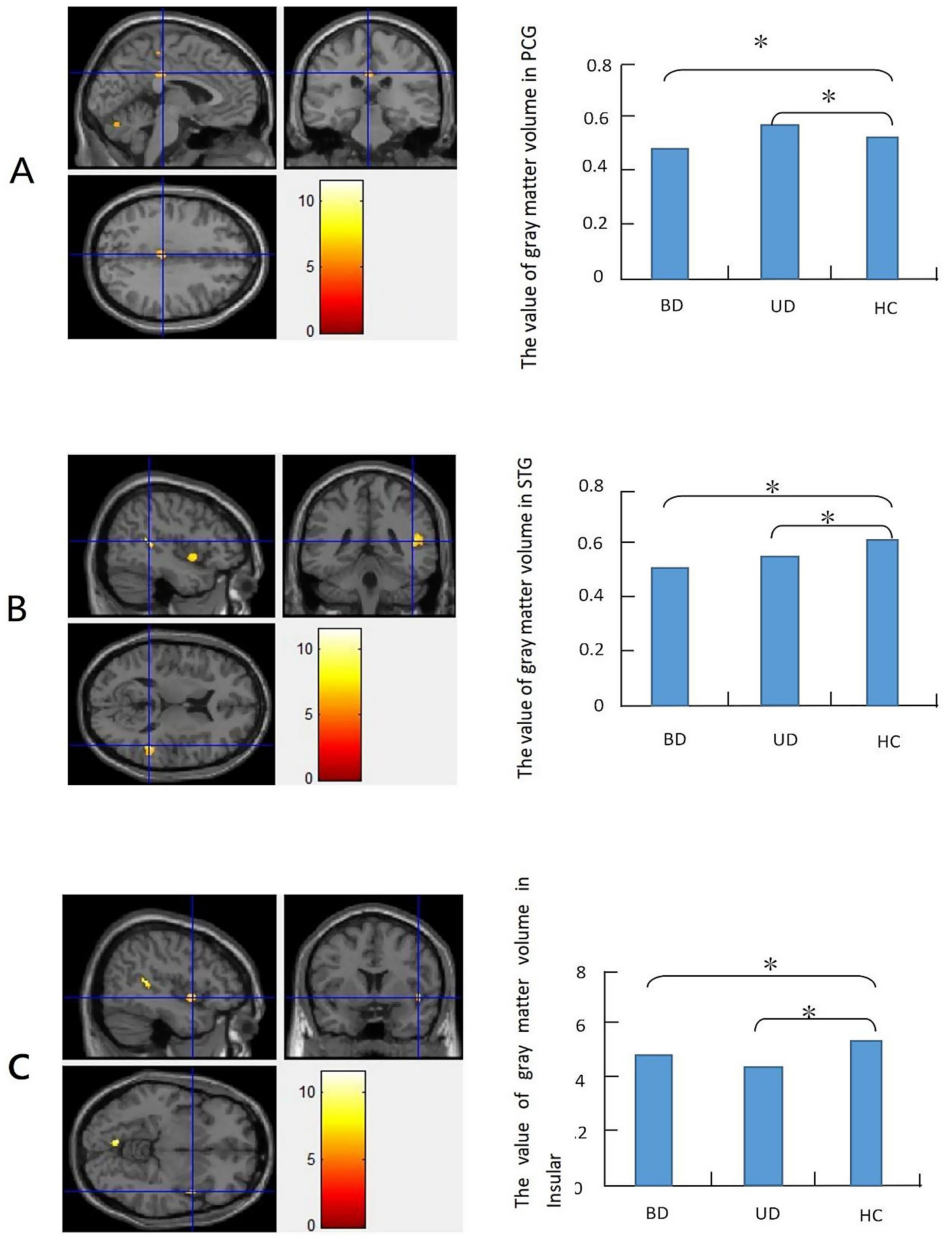
Panels **(A–C)** show comparisons of gray matter volume among the patients with bipolar depression (BD), those with unipolar depression (UD), and healthy controls (HCs). Statistically significant differences in gray matter volume were defined as a *p*-value of < 0.05 (FWE correction), with a cluster size of 35 adjacent voxels. The color bar represents the range of T values. Relative to the HCs, both patient groups showed significantly decreased gray matter volume in the right superior temporal gyrus (Panel **B**) and right insula (Panel **C**). The BD group exhibited decreased gray matter volume in the right posterior cingulate gyrus (Panel **A**), while the UD group showed increased gray matter volume in this region.

The one-way ANOVA results showed that brain areas with differences (*p* < 0.001, uncorrected) in gray matter volume were primarily located in the right insula, right superior temporal gyrus, and right posterior cingulate cortex. *Post-hoc t*-tests (FWE correction, *p* < 0.05) revealed that both patient groups showed significantly decreased gray matter volume in the right superior temporal gyrus and right insula compared to the healthy controls. Moreover, the BD group exhibited decreased gray matter volume in the right posterior cingulate cortex, while the UD group showed increased gray matter volume in this region (Table 2; Figure 1).

**Table 2:** Brain regions showing alterations in gray matter volume in the patients with bipolar depression (BD) and unipolar depression compared to the healthy controls.

Brain region	Cluster size (voxels)	Peak coordinates	Statistics
		X Y Z	*t p FWE, corrected*
BD<HCs			
Superior temporal gyrus	267	47, −40, 12	4.550.000283
Insula	46	47, 6, −6	4.620.000306
Posterior cingulate cortex	108	−3, −27, 33	5.02 0.000919
UD<HCs			
Superior temporal gyrus	249	51, −27, 9	5.010.000648
Insula	44	44, 11, −3	4.530.000273
UD>HCs			
Posterior cingulate cortex	94	2, −18, 9	5.040.000976

BD, bipolar depression; UD, unipolar depression; HCs, healthy controls; x, y, and z, coordinates of the primary peak locations in the Montreal Neurological Institute (MNI) space.

### Correlation between gray matter volume and depressive symptoms

As shown in Table 3, decreased gray matter volume in the right superior temporal gyrus (*r* = −0.033, *p* = 0.027) and right insula (*r* = −0.431, *p* = 0.044) was positively correlated with the HAMD scores in the UD group.

**Table 3:** Correlation between gray matter volume and HAMD scores in the UD group.

Item		Right superior temporal gyrus	Right insula
HAMD scores	*r*	−0.033	−0.431
	*p*	0.027	0.044

## Discussion

The main findings of the present study were as follows: there was significantly decreased gray matter volume in the right superior temporal gyrus and right insula in both patient groups compared to the HCs; there was a reduction in gray matter volume in the right posterior cingulate in the BD group; there was an increase in gray matter volume in this region in the UD group, compared to the HC group; and reductions in gray matter volume in the right superior temporal gyrus and right insula were positively associated with the total HAMD score.

In this study, we found that both patient groups showed significantly decreased gray matter volume in the right superior temporal gyrus and right insula compared to the HCs. The superior temporal gyrus, a part of the temporal lobe, showed decreased gray matter volume in patients with mood disorder (Beasley et al., 2005). The superior temporal gyrus is responsible for auditory processing and has connections with the amygdala and anterior cingulate cortex, which are thought to play a critical role in the processing and regulation of emotion and social cognition (Ma et al., 2007)_._ Moreover, the insula is a key structure in the “hate circuit,” which is involved in feelings of disgust and other emotions. Compared to the healthy controls, enhanced activation in the superior temporal gyrus in response to sad words and pictures was observed in the patients with BD (Mitchell et al., 2004), and decreased superior temporal gyrus volume in depression was negatively associated with the severity of depressive symptoms (Shah et al., 1998). In depressed patients, decreased basal activity in the insula and reduced responses to both positive and negative emotional stimuli, as well as reduced functional activity in the insula, have been demonstrated to be associated with the severity of depressive symptoms in fMRI studies. Moreover, the insula’s gray matter volume has been shown to be decreased in patients with BD or UD, irrespective of their mood states (Bechdolf et al., 2021; Brooks III et al., 2009; Peng et al., 2010; Sprengelmeyer et al., 2011). Therefore, the shared reduction in gray matter volume in these regions in this present study may help explain the overlapping clinical depressive symptoms between these two patient groups, as supported by our finding of significant associations between reduced gray matter volume and the HAMD scores.

This study found that the patients with BD specifically demonstrated decreased gray matter volume in the right posterior cingulate cortex, while the patients with UD exhibited increased gray matter volume in this region. The posterior cingulate cortex, an important region of the default mode network (DMN; Raichle et al., 2001), has connections with the amygdala and frontal cortex, which are thought to play a role in the regulation of emotion and cognitive functions in humans. Moreover, it is associated with top-down attentional control, which is involved in various cognitive processes, such as memory retrieval (Price and Drevets, 2012). Interestingly, our data showed that gray matter volume in the posterior cingulate cortex was reduced in the patients with BD but increased in the patients with UD. This finding is consistent with the clinical phenomenon that patients with BD experience more severe cognitive impairments compared to patients with UD. Consistently, compared to the healthy controls, the depressed patients showed enhanced activation in the posterior cingulate cortex in response to sad words. In addition, lesions in the posterior cingulate cortex have been shown to lead to deficits in memory retrieval. In depression, gray matter loss, functional abnormality, and inefficient activation during tasks in this region have been consistently documented to be related to depressive symptoms. Therefore, these data suggest that posterior cingulate cortex abnormalities may result from the emotional and cognitive deficits commonly observed in patients with depression. The structural changes in the posterior cingulate cortex (decreased gray matter volume) observed in UD may reflect a compensatory mechanism that helps maintain cognitive resilience.

In the present study, no abnormalities were detected in the gray matter nuclei of the prefrontal cortex and subcortical regions, such as the amygdala, hippocampus, and basal ganglia. This finding is not entirely consistent with the results of previous studies on mood disorders described in the introduction of this article. This discrepancy may be attributed to the following factors: First, the sample size of the patient groups (both depressive disorder subgroups) was relatively small, and the statistical threshold setting may have played a role. If the threshold was further relaxed, additional brain regions showing reduced gray matter volume in the patients (compared to the healthy controls) would include the parahippocampal gyrus and prefrontal cortex. Second, the mean duration of illness was 53.74 months in the bipolar depression group and 37.21 months in the unipolar depression group of this study; thus, the duration of illness may have influenced the results. In addition, some patients in our unipolar depression group might actually have bipolar depressive disorder but were misdiagnosed with unipolar depression because they had not experienced a manic episode. This limitation is difficult to avoid at the current stage and may have caused a certain degree of interference with the results of this study. We will conduct long-term follow-up of the patient groups. If misdiagnosed cases are identified, the data can be reanalyzed to obtain more robust results. In future studies, we will increase the sample size, enroll as many first-episode, medication-naive patients as possible, validate the findings of this study, and further explore the neuropathological mechanisms underlying unipolar and bipolar depressive disorders.

### Limitations

There are several limitations in this study that should be acknowledged. The BD group had a longer illness duration than the UD group. Therefore, we cannot fully rule out the possibility that our findings were influenced by this variable. Finally, previous studies have suggested that multivariate pattern analysis of brain images is a better approach for discriminating neuropsychiatric disorders. Future studies using multivariate pattern analysis of brain images may provide further insight into whether the distinct gray matter observed in this study is specific to BD or UD.

## Conclusion

In summary, in the present study, we demonstrated that different patterns of gray matter abnormalities in the right posterior cingulate cortex in bipolar depression and unipolar depression may be a biomarker for distinguishing BD from UD. This finding suggests that these different types of depression are involved in different neurological mechanisms, which may be valuable for clinical diagnosis and proper treatment choice for these two types of depression.

## Data Availability

The data analyzed in this study is subject to the following licenses/restrictions: no. Requests to access these datasets should be directed to liuchang19861121@163.com.

## Footnotes

1
http://www.fil.ion.uncl.ac.uk/spm

2
http://sourceforge.net/projects/s-fmri
