# Correction: Abnormalities of brain gray matter in bipolar depression and unipolar depression

**DOI:** 10.3389/fpsyg.2026.1765686

**Published:** 2026-03-30

**Authors:** Li Zhou, Guowei Wu, Xinchun Li, Chang Liu

**Affiliations:** 1 Department of Psychiatry, The Second People's Hospital of Hunan Province (Brain Hospital of Hunan Province), Changsha, China; 2 Second People's Hospital of Hunan Province, Yuhua, China; 3 Department of Psychiatry, First Affiliated Hospital of Jinan University, Guangzhou, Guizhou, China; 4 Mental Health Institute, Second Xiangya Hospital, Central South University, Changsha, Hunan, China

**Keywords:** depression, structural magnetic resonance imaging, gray matter volume, bipolar depression, unipolar depression

In the published article, there was an error in the affiliation sequence of the authors. The affiliation information was:

Li Zhou^1^, Guowei Wu^2^, Xinchun Li^3^ and Chang Liu^3, 4*^

1 Department of Psychiatry, First Affiliated Hospital of Jinan University, Guangzhou, Guizhou, China,

2 Mental Health Institute, Second Xiangya Hospital, Central South University, Changsha, Hunan, China,

3 Department of Psychiatry, The Second People's Hospital of Hunan Province (Brain Hospital of Hunan Province), Changsha, China,

4 Second People's Hospital of Hunan Province, Yuhua, China

The correct affiliation information should be:

Li Zhou^2^, Guowei Wu^3^, Xinchun Li^1^ and Chang Liu^1*^

1 Department of Psychiatry, The Second People's Hospital of Hunan Province (Brain Hospital of Hunan Province), Changsha, China,

2 Department of Psychiatry, First Affiliated Hospital of Jinan University, Guangzhou, Guizhou, China,

3 Mental Health Institute, Second Xiangya Hospital, Central South University, Changsha, Hunan, China

The original version of this article has been updated.

